# Demographic and regional trends of mortality in patients with cardiovascular disease and liver cirrhosis in the United States between 1999 and 2019

**DOI:** 10.1016/j.athplu.2025.09.004

**Published:** 2025-09-20

**Authors:** Farah Yasmin, Abdul Moeed, Muhammad Ahmed Ali Fahim, Gaurav Kumar, Maryam Shaharyar, Muhammad Sohaib Asghar

**Affiliations:** a Yale School of Medicine, New Haven, CT, USA; b Dow University of Health Sciences, Karachi, Pakistan; c Mayo Clinic, Rochester, MN, USA; d AdventHealth, Sebring, FL, USA

## Abstract

**Objectives:**

National estimates of deaths related to cardiovascular disease (CVD) and liver cirrhosis remain ambiguous. The purpose of this study was to observe the contemporary trends in CVD and liver cirrhosis-related mortality in the United States.

**Methods:**

We evaluated the trends using the CDC WONDER database to identify adults with CVD and liver cirrhosis associated death between 1999 and 2019. Age-adjusted mortality rates (AAMRs) per 100,000 population and associated average annual percent changes (AAPCs) with 95 % confidence intervals (CIs) were assessed using Joinpoint regression.

**Results:**

Between 1999 and 2019, a total of 374,090 deaths occurred due to CVD and liver cirrhosis. In the overall population, the AAMR increased from 7.54 (95 % CI 7.41–7.67) in 1999 to 10.55 (95 % CI 10.43–10.68) with an AAPC of 1.68 (95 % CI 1.59–1.77). The highest AAMRs were seen in males, Native Americans, and those living in West. A substantial increase in AAMRs was observed in all age groups with the highest seen in 70–84 year group. Moreover, non-metropolitan cities had a much higher increase in AAMRs compared to large metropolitan cities. The highest AAMRs were observed in California whereas the lowest in Utah.

**Conclusion:**

There is a rising trend of CVD and liver cirrhosis-associated mortality in all groups. However, disparities continue to exist in association with gender, race, age, and geographical region. Future trials should address alleviating CVD and liver cirrhosis-related deaths in all population groups equitably.

## Highlights

• We evaluated the trends in national estimates of deaths related to cardiovascular disease (CVD) and liver cirrhosis between 1999 and 2019
• The highest AAMRs were observed in California whereas the lowest in Utah.
• There is a rising trend of CVD and liver cirrhosis-associated mortality in all groups. However, disparities continue to exist in association with gender, race, age, and geographical region.

## Graphical abstract

**Figure:**
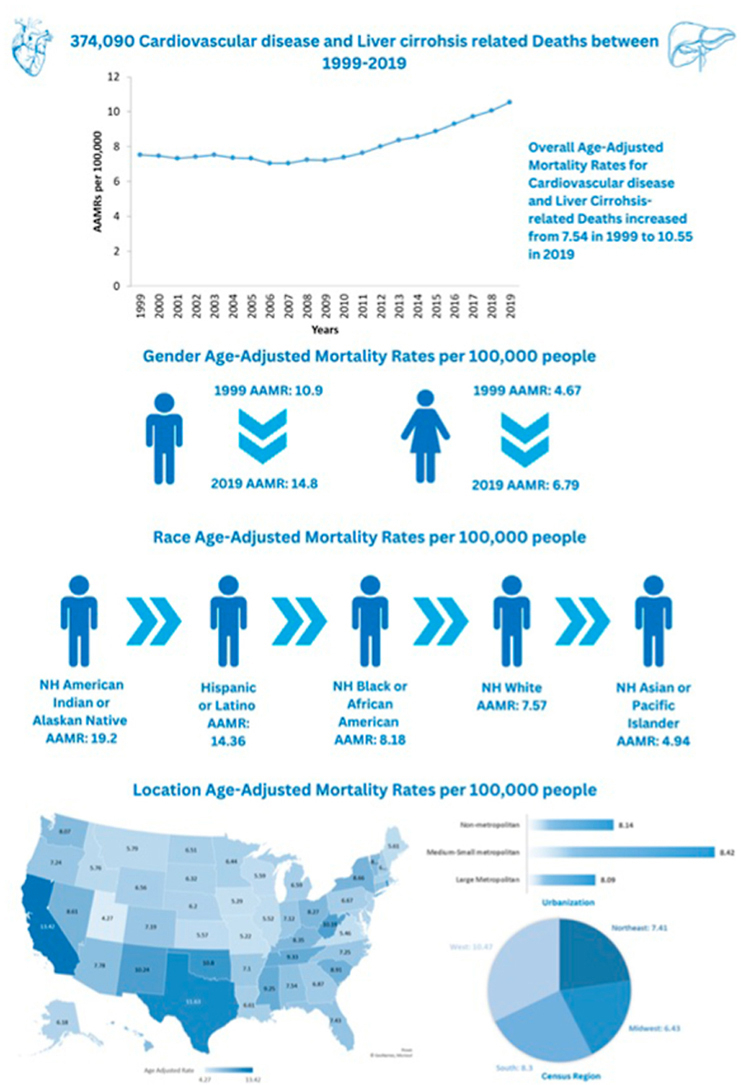

## 1 Introduction

Cardiovascular disease (CVD) is the leading cause of death in the United States (US), accounting for 26.9 % of yearly deaths across all age groups [1]. Similarly, liver cirrhosis remains a significant contributor to mortality [2], with rates projected to increase due to the increasing prevalence of alcoholic liver disease and Metabolic Dysfunction- Associated Steatotic Liver Disease (MASLD) [3,4]. Numerous scientific studies have demonstrated a strong association between CVD and liver disease, both of which share many common risk factors such as dyslipidemia, diabetes mellitus, and obesity, creating a complex interplay that exacerbates their burden on public health and economy [[5], [6], [7]].

The pathophysiological connection between liver cirrhosis and CVD is well-documented [8]. Portal hypertension in a cirrhotic liver distresses the heart via peripheral and splanchnic arterial vasodilation, establishing a hyperdynamic circulation with increased cardiac output and heart rate and reduced arterial blood pressure, that eventually contributes to the development of “cirrhotic cardiomyopathy” [[9], [10], [11]]. Bacterial translocation, systemic hepatic inflammation, and increased bile acid levels in the blood in cirrhosis also induces cardiac remodeling via release of inflammatory mediators [12,13]. This damage to the cardiac structure and physiology by a diseased liver is independent of cirrhosis etiology, which ranges from chronic infections with hepatitis B virus (HBV) or hepatitis C (HCV) virus, heavy and chronic alcoholic intake developing alcoholic liver disease, and the inflammatory MASLD [14]. Conversely, the hemodynamic changes that accompany heart failure may also damage liver function and structure, illustrating a bidirectional relationship between these diseases [15,16].

Considering these interactions between liver cirrhosis and CVD, the evaluation of both these diseases concurrently is of emerging scientific interest. Cardiovascular complications are a major cause of death in patients with MASLD but real-world data on coincident CVD and cirrhosis burden, especially in other etiologies of cirrhosis, is sparse. Therefore, this study aims to address this gap by analyzing regional and demographic trends in mortality rates due to CVD and liver cirrhosis among US. Adults from 1999 to 2019.

## 2 Methods

### 2.1 Study setting and population

The Centers for Disease Control and Prevention Wide-ranging OnLine Data for Epidemiologic Research (CDC-WONDER) database was examined to retrospectively obtain data on Liver cirrhosis and CVD-related mortality in the US between 1999 and 2019. The Multiple Cause-of-Death dataset was analyzed to identify instances where Liver cirrhosis and CVD were listed as either underlying or contributing causes of death [17]. The International Classification of Diseases and Related Health Problems −10th Revision (ICD-10) codes were used to identify relevant death certificates with liver cirrhosis classified under Alcoholic (K70.3), Toxic (K71.7), and other liver cirrhosis (K74), while CVD was identified using codes I00-I99. Individuals aged 25 years or older at the time of death were included in our analysis. As publicly available, deidentified death record data was utilized Institutional Review Board approval was not required. Moreover, our study adhered to the Strengthening the Reporting of Observational Studies in Epidemiology (STROBE) guidelines for rigorous reporting standards.

### 2.2 Data abstraction

Data on deaths due to concomitant liver cirrhosis and CVD was categorized based on year of death, location of death, demographics, urbanization, region, and state. Location of death was further subclassified into medical facilities (including outpatient, emergency room, inpatient, death on arrival, or status unknown), decedents' home, hospice, and nursing home or long-term care facility. Similarly, decedent demographics included gender, age and race, with

race subcategories including non-Hispanic (NH) White, NH Black or African American, Hispanic or Latino, NH American Indian or Alaskan Native and NH Asian or Pacific Islander. This classification of race follows the US Office of Budget and Management guidelines; furthermore, it has also been used by previous studies examining the database [18]. The National Center for Health Statistics Urban-Rural Classification Scheme was used to stratify the population by large metropolitan area (≥1,000,000 people), medium-small metropolitan area (50,000–999,999 people), and non-metropolitan area (<50,000 people) counties in accordance with the 2013 U S. Census classification [19]. Additionally, regions were classified into North, Midwest, South, and West based on the U.S. Census Bureau's characterizations [20].

### 2.3 Statistical analysis

To analyze trends in mortality and ensure compatibility across different subgroups, we calculated crude and Age-Adjusted Mortality Rates (AAMRs) per 100,000 people with their respective 95 % Confidence Intervals (CI). AAMRs were standardized to the 2000 U S. Population while crude mortality rates were determined by dividing the number of liver cirrhosis and CVD-related deaths by the corresponding US population of that year [21]. To identify and analyze trends, Annual Percentage Change (APC) in AAMRs was calculated through the Joinpoint Regression Program (Joinpoint V 5.0.2, National Cancer Institute) [22]. This software makes use of log-linear regression models to detect significant changes in AAMR over time. This method enables identification of statistically significant changes in temporal trends, including discontinuity points (i.e., joinpoints) and slope changes. The software applies permutation tests to assess whether observed changes represent true inflection points rather than empirical fluctuations. Using 2‐tailed t-testing, APCs were considered increasing or decreasing if the change in mortality was significantly different from zero. Results were considered to be statistically significant if P < 0.05.

## 3 Results

From 1999 to 2019, CVD and liver cirrhosis were responsible for 374,090 deaths in patients aged 25 years and above (Supplementary Table 1). 65.94 % of deaths were male with NH Whites (69.83 %) and those aged between 55 and 69 years (40.80 %) having the highest proportional mortality. Geographically, Large metropolitan areas (51.98 %) and the South (37.45 %) had the highest mortality count. When stratified by place of death, medical facilities accounted for 55.54 % of deaths, decedents' homes for 25.67 %, nursing homes for 10.80 %, and hospices for 3.89 %. Moreover, 3.87 % of deaths occurred at places that could not be classified into the aforementioned categories, with the place of death for the remaining 846 patients remaining unknown. (Supplementary Table 2). The overall AAMR for all age groups was 8.2 (95 % CI: 8.17–8.22). Analyzing crude rates revealed that those aged between 70 and 84 years had the highest rates (21.7) followed by 55–69 years (15.4), 40–54 years (6.19), and lastly 25–39 years (0.67) Demographic data is provided in Table 1.

**Table 1:** Demographic characteristics of deaths due to liver cirrhosis and cardiovascular disease in adults in the United States, 1999 to 2019.

Variable	Chronic Liver Disease and Cardiovascular Disease Deaths n (%)	Age Adjusted Mortality Rates/Crude Rates (95 % CI) per 100,000
**Overall Population**	374090	8.2 (8.17–8.22)
**Sex**		
Male	246662 (65.94)	11.72 (11.67–11.76)
Female	127428 (34.06)	5.14 (5.11–5.17)
**Age**		
25–39 years	8853 (2.37)	0.67 (0.66–0.69)^a^
40–54 years	82737 (22.12)	6.19 (6.15–6.23)^a^
55–69 years	152623 (40.80)	15.4 (15.32–15.48)^a^
70–84 years	106935 (28.59)	21.7 (21.57–21.83)^a^
85+ years	22942 (6.13)	20.33 (20.07–20.59)^a^
**Census Region**		
Northeast	64447 (17.23)	7.41 (7.35–7.47)
Midwest	64730 (17.30)	6.43 (6.38–6.48)
South	140083 (37.45)	8.3 (8.26–8.35)
West	104830 (28.02)	10.47 (10.41–10.54)
**Race/Ethnicity**		
NH American Indian or Alaska Native	5764 (1.54)	19.2 (18.69–19.72)
NH Asian or Pacific Islander	8863 (2.37)	4.94 (4.83–5.04)
NH Black or African American	39220 (10.48)	8.18 (8.1–8.27)
NH White	261210 (69.83)	7.57 (7.54–7.6)
Hispanic or Latino	57485 (15.37)	14.36 (14.24–14.49)
**Urbanization**		
Large Metropolitan	194459 (51.98)	8.09 (8.06–8.13)
Medium-Small metropolitan	117212 (31.33)	8.42 (8.37–8.47)
Non-metropolit^a^n	62419 (16.69)	8.14 (8.07–8.2)
**Place of Death**^b^		
Medical Facility	207758 (55.54)	–
Decedent's Home	96017 (25.67)	–
Hospice Facility	14534 (3.89)	–
Nursing Home/Long-term Care Facility	40396 (10.80)	–
Others	14494 (3.87)	–
Unknown	846 (0.23)	–

NH: Non-Hispanic.

a Crude Rates are calculated for age groups.

b Age-Adjusted Mortality Rates (AAMRs) are not applicable for Place of Death.

### 3.1 Annual trends for cardiovascular disease and liver cirrhosis -related Age-Adjusted Mortality Rates

**Fig. 1:**
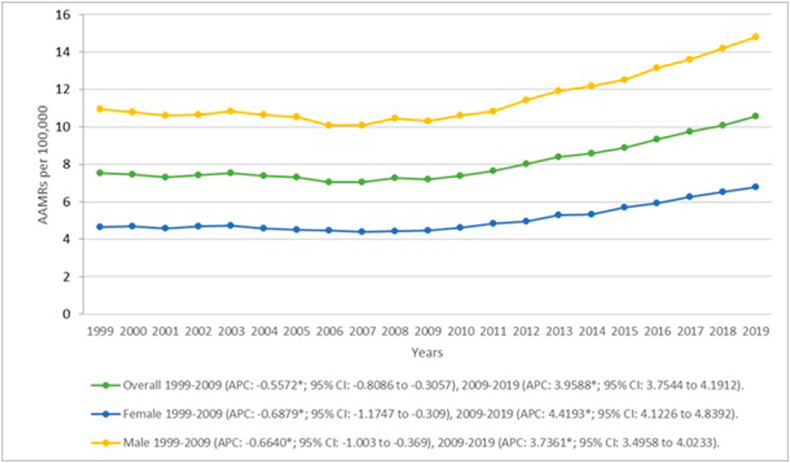
Overall and sex-stratified liver cirrhosis and cardiovascular disease-related AAMRs per 100,000 in the United States, 1999 to 2019.

Overall, AAMRs for patients with CVD and liver cirrhosis decreased significantly in the initial years from 7.54 (95 % CI: 7.41–7.67) in 1999 to 7.21 (95 % CI: 7.1–7.33) in 2009 (APC: −0.5572*; 95 % CI: −0.8086 to −0.3057). This was followed by a significant increase, with rates reaching 10.55 (95 % CI: 10.43–10.68) by 2019 (APC: 3.9588*; 95 % CI: −3.7544 to 4.1912). (Fig. 1, Supplementary Tables 3 and 4).

### 3.2 Cardiovascular disease and liver cirrhosis -related Age-Adjusted Mortality Rates stratified by sex

When analyzing mortality by gender, males were found to have notably higher AAMRs than females throughout the study period (total AAMR male: 11.72; 95 % CI: 11.67–11.76 vs total AAMR female: 5.14; 95 % CI: 5.11–5.17) with rates decreasing significantly from 10.94 (95 % CI: 10.71–11.17) to 10.3 (95 % CI: 10.1–10.5) between 1999 and 2009 respectively (APC-0.6640*; 95 % CI: −1.003 to −0.369) and consequently showing a significant increase to 14.8 (95 % CI: 14.58–15.02) in 2019. (APC: 3.7361*; 95 % CI: 3.4958 to 4.0233) Similarly, females also presented a significant decrease in the initial years with AAMRs falling from 4.67 (95 % CI: 4.54–4.81) to 4.48 (95 % CI: 4.36–4.6) between 1999 and 2009 (APC: −0.6879*; 95 % CI: −1.1747 to −0.309) with an increase to 6.79 (95 % CI: 6.65–6.93) by 2019 that attained statistical significance (APC: 4.4193*; 95 % CI: 4.1226 to 4.8392). (Fig. 1, Supplementary Tables 3 and 4).

### 3.3 Cardiovascular disease and liver cirrhosis -related Age-Adjusted Mortality Rates stratified by race

**Fig. 2:**
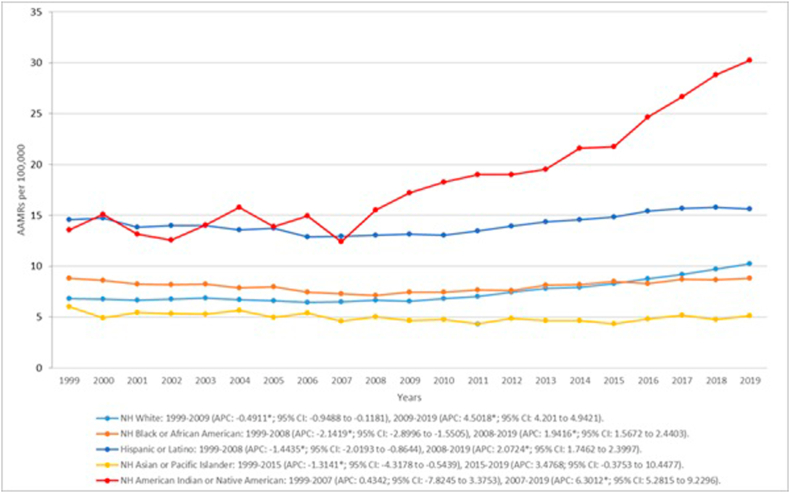
Liver cirrhosis and cardiovascular disease-related AAMRs per 100,000 stratified by race in the United States, 1999 to 2019.

Stratifying by race AAMRS were highest in NH American Indians or Native Americans (AAMR:19.2; 95 % CI: 18.69–19.72), Hispanics or Latinos (AAMR:14.36; 95 % CI:14.24–14.49), NH Black or African American (AAMR:8.18; 95 % CI: 8.1–8.27), NH White (AAMR:7.57; 95 % CI: 7.54–7.6), NH Asian or Pacific Islander (AAMR:4.94; 95 % CI:4.83–5.04). For NH American Indians or Native Americans AAMRs remained stable from 1999 to 2007 showing little change (APC: 0.4342; 95 %CI: −7.8245 to 3.3753). However, from this point forward a drastic increase in AAMRs was noted by 2019 that attained significance on analysis (APC: 6.3012*; 95 %CI: 5.2815 to 9.2296). Both the Hispanic or Latino (APC: −1.4435*; 95 %CI: −2.0193 to −0.8644) and NH Black or African Americans (APC: −2.1419*; 95 %CI: −2.8996 to −1.5505) populations showed a significant decrease in mortality till 2008 after which both populations exhibiting a drastic increase till the end of the study period. (NH Black or African American APC: 1.9416*; 95 %CI: 1.5672 to 2.4403) (Hispanic or Latino APC: 2.0724*; 95 %CI: 1.7462 to 2.3997). Trends for NH White mimicked overall trends with a fall in mortality between 1999 and 2009 (APC: −0.4911*; 95 %CI: −0.9488 to −0.1181) followed by a surge in deaths till the end of the study period. (APC: 4.5018*; 95 %CI: 4.201 to 4.9421). Contrastingly NH Asian or Pacific Islander exhibited a decline in mortality rates till 2019 (APC: −1.3141*; 95 %CI: −4.3178 to −0.5439) after which rates remained relatively stable. (APC: 3.4768; 95 %CI: −0.3753 to 10.4477). (Fig. 2, Supplemental Tables 3 and 5).

### 3.4 Cardiovascular disease and liver cirrhosis -related Age-Adjusted Mortality Rates stratified by geography

**Fig. 3:**
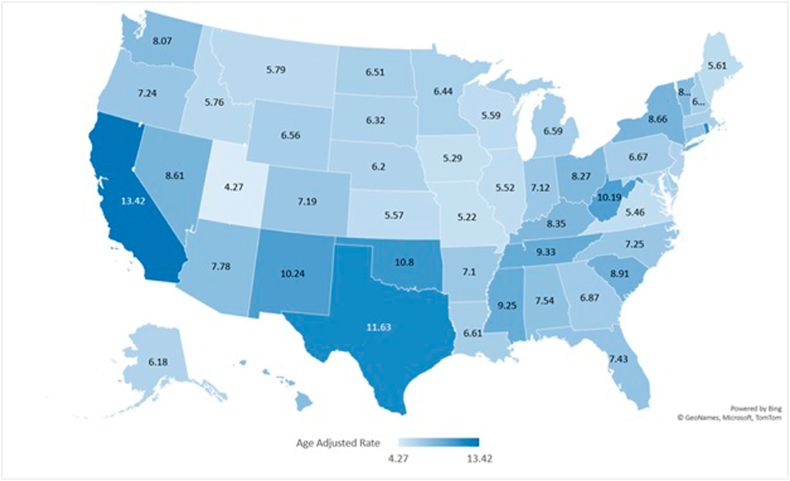
Liver cirrhosis and cardiovascular disease-related AAMRs per 100,000 stratified by state in the United States, 1999 to 2019.

Evaluating state-wise data showed AAMRs ranging from 13.42 (95 % CI: 13.32–13.52) in California to 4.27 (95 % CI: 4.03–4.5) in Utah with states in the top 90th percentile (California, Texas, Rhode Island, Oklahoma and New Mexico) having over triple the mortality rates than those in the lower 10th percentile (Illinois, Virginia, Iowa, Missouri and Utah). (Fig. 3 Supplemental Table 6).

**Fig. 4:**
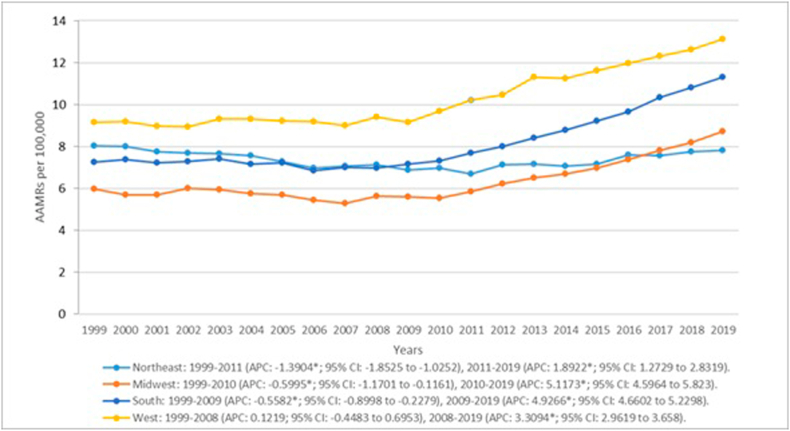
Liver cirrhosis and cardiovascular disease-related AAMRs per 100,000 stratified by census region in the United States, 1999 to 2019.

According to census regions, the West presented with the highest overall AAMR at 10.47 (95 % CI: 10.41–10.54) followed by the South at 8.3 (95 % CI: 8.26–8.35), the Northeast at 7.41 (95 % CI: 7.35–7.47) and the Midwest at 6.43 (95 % CI: 6.38–6.48). (Fig. 4, Supplemental Table 7). The Northeast revealed AAMRs that decreased significantly between 1999 and 2011 (APC: −1.3904*; 95 %CI: −1.8525 to −1.0252) which was followed by a significant increase till 2019 (APC: 1.8922*; 95 %CI: 1.2729 to 2.8319). AAMRs for decedents in the Midwest decreased slightly between 1999 and 2010 (APC: −0.5995*; 95 %CI: −1.1701 to −0.1161) but saw a substantial increase over the next nine years (APC: 5.1173*; 95 %CI: 4.5964 to 5.823). A similar trend was observed in the South with slight significant decreases in the initial half of the study from 1999 till 2009 (APC: −0.5582*; 95 %CI: −0.8998 to −0.2279) superseded by a notable increase in the latter half till 2019 (APC: 4.9266*; 95 %CI: 4.6602 to 5.2298). Contrastingly rates in the West remained stable from 1999 till 2008 (APC: 0.1219; 95 %CI: −0.4483 to 0.6953) and increased significantly from 2008 till 2019 (APC: 3.3094*; 95 %CI: 2.9619 to 3.658).

### 3.5 Cardiovascular disease and liver cirrhosis -related Age-Adjusted Mortality Rates stratified by urbanization

**Fig. 5:**
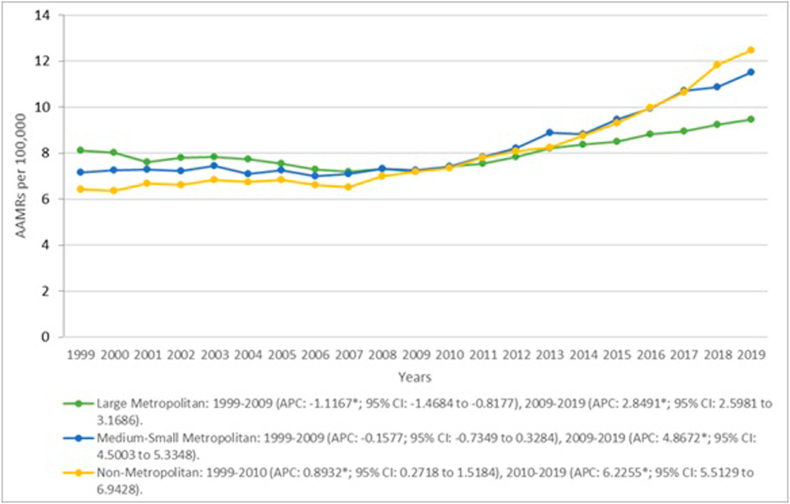
Liver cirrhosis and cardiovascular disease-related AAMRs per 100,000 stratified by urbanization in the United States, 1999 to 2019.

When analyzing data according to urbanization Medium-Small metropolitan areas at 8.42 (95 % CI: 8.37–8.47), had the highest overall AAMR followed by Non-metropolitan at 8.14 (95 % CI: 8.07–8.2), and Large metropolitan regions at 8.09 (95 % CI: 8.06–8.13). Mortality rates significantly declined in the initial ten years in Large metropolitan areas from 8.11 (95 % CI: 7.93–8.29) in 1999 to 7.19 (95 % CI: 7.03–7.35) in 2009 (APC: −1.1167*; 95 % CI: −1.4684 to −0.8177) increasing to 9.48 (95 % CI: 9.31–9.64) in 2019 (APC: 2.8491*; 95 % CI: 2.5981 to 3.1686). Contrastingly, Medium-Small metropolitan areas showed an initial trend of stable AAMRs between 7.17 (95 % CI: 6.95–7.4) and 7.26 (95 % CI: 7.05–7.47) from 1999 to 2009 (APC: −0.1577; 95 % CI: −0.7349 to 0.3284) with mortality rates rising drastically to 11.51 (95 % CI: 11.27–11.75) by the end of the study period (APC: 4.8672*; 95 % CI: 4.5003 to 5.3348). Additionally, for nonmetropolitan areas a significant increase in mortality rates was evident from 6.42 (95 % CI: 6.14–6.7) in 1999 to 7.36 (95 % CI: 7.07–7.64) in 2010 (APC: 0.8932*; 95 % CI: 0.2718 to 1.5184) which escalated to 12.46 (95 % CI: 12.1–12.82) by 2019 (APC: 6.2255*; 95 % CI: 5.5129 to 6.9428). (Fig. 5, Supplemental Tables 3 and 8).

## 4 Discussion

This 20-year analysis of mortality data from the CDC WONDER database yielded several key findings. Firstly, after an initial period of mild decline from 1999 to 2009, mortality due to cirrhotic liver disease and cardiovascular disease increased significantly for the rest of our study period. The same trend followed suit when stratified by sex, with men having consistently higher AAMRs than women throughout the study period. NH American Indians or Native Americans had the highest AAMR which showed a significant increase post-2007 after a period of stability. While every racial group's trends aligned with the overall population pattern, NH Asian or Pacific Islanders exhibited a decline in mortality till 2019. Fourth, notable regional disparities were observed with states in the 90th percentile (California, Texas, Rhode Island, Oklahoma, and New Mexico) having AAMRs threefold higher than those in the bottom 10th percentile (Illinois, Virginia, Iowa, Missouri, and Utah). These results bear crucial significance for public health initiatives and future research.

The shifts in mortality due to cirrhosis and CVD highlight the changing distribution of the underlying etiologies of cirrhosis. Increased HBV vaccination coverage and improved availability of antivirals have significantly reduced rates of HBV- and HCV-associated cirrhosis [2,23,24]. Although alcohol consumption has risen dramatically over the past two decades [25,26], death rates for alcohol-related cirrhosis have declined [27], though concerns regarding underreporting and underdiagnosis persist. On the contrary, the growing prevalence of obesity and diabetes has fueled a rapid increase in the prevalence of MASLD-associated cirrhosis [27]. Incident cirrhosis is projected to increase till 2040 [28], largely driven by MASLD which shares the same risk factors as CVD. While our dataset did not allow for direct analysis of liver disease etiology, it is important to interpret mortality trends in the context of shifting causes of liver cirrhosis in the U.S. There has been a marked rise in MASLD-related liver disease, now recognized as a leading cause of cirrhosis and liver-related mortality. Alcohol-associated liver disease (ALD) also remains a significant contributor and may confound observed trends, especially given increasing rates of alcohol use disorder in certain subpopulations during the study period. Future studies incorporating etiology-specific data are warranted to delineate these patterns more precisely. In addition to etiologic heterogeneity, external factors such as diet, environmental exposures, and urbanization may also contribute to the observed epidemiological trends. Studies from international settings suggest that dietary patterns, air pollution, and regional socioeconomic status influence both liver and cardiovascular disease burdens. These exposures may intersect with geographic, racial, and census-related patterns within the U.S., and warrant further investigation.

Our study demonstrates consistently higher mortality rates in men due to CVD and cirrhosis as compared to women. CVD is significantly more prevalent in men as compared to women because despite sharing common risk factors, age, hypertension, total cholesterol, and low-density lipoprotein (LDL)-cholesterol reportedly has a greater influence in men [29]. On the contrary, women have a higher rate of mortality and poor prognosis following an acute cardiovascular event [30]. Furthermore, a meta-analysis by Khalid YS et al. reported a twofold increase in cardiovascular events and mortality in females with MASLD [31]. Alcohol consumption has also seen an upward trajectory in women in recent decades, raising concerns given their increased susceptibility to alcoholic liver disease with a lower alcohol intake [32].-^33^ Despite this, the gender disparity in mortality rates of concomitant CVD and cirrhosis is consistent with that observed for cirrhosis alone [32]. Interestingly, the rate of increase in mortality among younger adults is higher in women when compared with men, but that disparity is driven by race, especially among NH White and Hispanic individuals [33].

Race-based differences in AAMRs for CVD and cirrhotic liver disease align with existing literature, emphasizing a higher burden of disease among American Indians and Native Americans. A CDC-WONDER analysis of cirrhosis mortality from 1999 to 2016 reflected the highest rates for American Indian or Alaska Native individuals, followed by Latino, White, Black, and Asian or Pacific Islanders [34]. These differences persist when CVD is taken into account and can be alluded to the relative prevalence and racial differences in the underlying etiologies of cirrhosis. For example, Rich NE et al.’s meta-analysis reported a higher prevalence of MASLD in the Latino population (22.9 %) than the White (14.4 %) and Black (13.0 %) population [35], driven by differences in exposure to metabolic comorbidities and risk factors [36]. A lower visceral fat deposition in Black individuals is associated with a milder course of MASLD due to reduced liver damage and inflammation [37,38]. Similarly, National Health and Nutrition Examination Survey data revealed a high prevalence of alcoholic liver disease in the American Indian or Alaska Native and Latino population, which contributes to the observed disparities in mortality due to cirrhosis and CVD [39]. Alcoholic liver disease is the leading cause of cirrhosis-related mortality in American Indian or Alaska Native and NH Whites [32,40], likely driven by the increased use of recreational drugs and alcohol in this group [41]. Chronic HCV infection, a major cause of cirrhosis, also shows a poor prognosis in American Indian or Alaska Native individuals when compared to the rest of the population [42]. In a study of newly diagnosed patients with HCV, diabetes, and obesity, the adjusted odds ratio (aOR) of advanced liver disease was nearly 8 times higher (aOR, 7.89; 95 % CI, 3.66–17.01) for Hispanic versus Black patients, and 12 times higher (aOR, 12.49; 95 % CI, 3.24–48.18) for Hispanic versus White patients [43].

Patients from underrepresented ethnic and racial groups face higher barriers to diagnosis and care of both CVD and cirrhosis, receive less optimal care, and experience worse outcomes than their White counterparts [44]. Among the American Indian or Alaska Natives, Black, and Latino populations, structural discrimination and long-standing policies have led to higher exposure to disease risk factors, fewer economic opportunities, lower educational development, and social isolation that puts them at increased risk [45].-^46^ Prior studies have demonstrated that Black patients with CVD are less likely to undergo transplantation [47], have a higher risk of death post-transplant, and a lower probability of receiving anticoagulation or other interventional therapies as opposed to white patients [48]. Similarly, Nephew LD's analysis of the National Inpatient Sample yielded lower odds of receiving complicated, high-risk procedures such as Trans-jugular Intrahepatic Portosystemic Shunt and liver transplant in hospitalized cirrhosis patients than their white counterparts [49]. Black and Latino individuals have also been found to receive fewer referrals for HCV surgical procedures and HCV treatment for end-stage liver disease [46]. Additionally, Hispanic participants have significantly lower access to alcohol and substance use treatment than White patients, explaining their higher predisposition to alcoholic cirrhosis [50]. Addressing these disparities requires targeted efforts to improve access to care, eliminate systemic barriers, and promote equity in healthcare delivery [51].

The observed racial and ethnic disparities in CVD and LC mortality may, in part, reflect underlying genetic differences [52]. Polymorphisms affecting insulin sensitivity, lipid metabolism, hepatic steatosis, and fibrosis progression have been shown to vary across racial groups [53]. For example, the *PNPLA3* I148M variant, associated with hepatic fat accumulation and fibrosis, is more prevalent among Hispanic populations [54]. However, genetic predisposition alone is unlikely to explain the extent of disparity. Structural factors such as healthcare access, socioeconomic status, and exposure to systemic racism likely amplify these genetic risks, underscoring the need for multifactorial models in disparity research [55].

Regional variations in cirrhosis mortality have also been observed, with notably high rates occurring in the South and West [56].-^57^ When our study accounts for CVD as well, Southwest (California, Texas, and New Mexico) and Northeast (Rhode Island) regions are also concerning. Similarly, mortality due to CVD is also the highest in the Deep South, besides the Appalachia, as noted by Rao S et al. [58] Conversely, the West, Midwest, and Northwestern regions report the lowest CVD mortality rates, largely attributable to lower proportions of Black populations, higher educational attainment, lower rates of smoking and obesity, and higher median household incomes [58]. For end-stage liver disease, longer travel distances to liver specialists and liver transplant centers contribute to higher mortality. For example, as of 2024, New Mexico does not have a single liver transplant center [59]. Additionally, variations in public health policy over major contributors to chronic liver disease such as alcohol consumption to sociocultural and religious attitudes and HCV further explain these disparities [57]. An analysis by Desai AP et al. identified Hispanic ethnicity and median household income as key factors influencing geographic variation in liver disease mortality, ruling out obesity distribution as a possible explanation [57]. Socioeconomic status (SES) remains one of the strongest determinants of CVD [[60], [61], [62]], explaining major disparities in outcomes by ethnicity and rate in the USA. Similarly, MASLD burden varies by SES as well, whereby limited access to nutritional food is associated with higher odds of developing MASLD and associated cirrhosis [63]. Indicators such as income, wealth, employment status, occupation, and housing conditions underlie the rural-urban disparities observed in our study.

Although our study provides a comprehensive overview of mortality trends, there are several limitations to consider when interpreting the findings. With the use of ICD-10 codes and death certificates to determine the cause of death, there exists a potential for misclassification bias. Additionally, valuable information such as that of competing risks of death, investigations, treatments, procedures, laboratory parameters and patient socioeconomic background could not be attained due to the inherent limitations of the CDC WONDER database. Moreover, our study does not specify the CVD subtypes included. Lastly, the recorded place of death and the patient's actual residence might differ, potentially introducing bias in geographical analyses.

## 5 Future recommendations

The results of our analysis of cirrhosis and CVD mortality bear crucial significance for public health initiatives and future research. Clinicians should consider a multi-disciplinary evaluation of these two diseases which share many of the same risk factors such as dyslipidemia, obesity, and diabetes mellitus. Collaboration of primary care physicians with cardiologists and hepatologists to conduct a holistic evaluation of at-risk patients, especially NH American Indians or Native Americans, in multispecialty clinics is bound to alleviate the burden these diseases place on public health and the economy. There is a pressing need at the grassroots level to view these two diseases in tandem with each other, rather than separately, to reduce disease progression, improve health outcomes, and prevent mortality. This goes for physicians, as well as researchers working to develop novel therapeutic drugs. We need more headway in understanding the molecular mechanisms by which the hepatic and cardiovascular systems interact, so that any new therapy accounts for this synergistic relationship.

The geographical granularity of these findings not only serves as a potential roadmap for future analysis of the drivers of these trends but also for the design and implementation of more effective, targeted interventions. For example, understanding the variations in alcohol consumption, smoking, socioeconomic status, and obesity across the US is the first step towards proposing targeted counseling, product marketing, and legislation by the healthcare and the government. Rural areas and regions such as the South and West with the highest cirrhosis mortality need better access to hepatologists and liver transplant centers, as evidenced by the lack of any in New Mexico. Strategies to recruit specialist physicians to these regions must also be developed.

Our study outlines the trends until 2019 and while these findings are crucial, predictive models and longitudinal studies should evaluate trends that go beyond. The impact of the COVID-19 pandemic affected mortality rates across the board, which is why studying its impact on particularly CVD and cirrhosis mortality is of great scientific interest. Furthermore, confounders such as socioeconomic indicators, behavioral risk factors, lifestyle, biomarkers, and laboratory markets should be considered in any further research observing the interplay between cirrhosis and CVD mortality.

## 6 Conclusion

Our study provides valuable insight into liver cirrhosis and CVD-related mortality in the US over two decades, which has shown an overall increase. Death rates peaked in males, individuals of NH American Indian or Alaskan Native descent, and residents of California, the West, and Medium-small metropolitan areas. Targeted interventions tailored to local and individual needs are necessary to address the apparent disparities, with further trials being crucial to establish a clear causal association.

## Declaration of competing interest

The authors declare that they have no known competing financial interests or personal relationships that could have appeared to influence the work reported in this paper.
