# Supplementary material for: Demographic and regional trends of mortality in patients with cardiovascular disease and liver cirrhosis in the United States between 1999 and 2019
Source: Atheroscler Plus. 2025 Sep 20;62:1–8. doi: 10.1016/j.athplu.2025.09.004 (PMC12492282; doi:10.1016/j.athplu.2025.09.004)
Supplement: Multimedia component 1 [file mmc1.docx]

| **Year** | **Overall** | **Women** | **Men** | **NH White** | **NH Black or African American** | **NH Asian or Pacific Islander** | **NH American Indian or Alaska Native** | **Hispanic or Latino** | **Population** |
| --- | --- | --- | --- | --- | --- | --- | --- | --- | --- |
| 1999 | 13343 | 4653 | 8690 | 9766 | 1501 | 288 | 135 | 1596 | 180408769 |
| 2000 | 13462 | 4737 | 8725 | 9780 | 1505 | 246 | 157 | 1706 | 181984640 |
| 2001 | 13468 | 4682 | 8786 | 9747 | 1498 | 297 | 148 | 1725 | 184305128 |
| 2002 | 13862 | 4847 | 9015 | 9987 | 1536 | 298 | 145 | 1827 | 186208028 |
| 2003 | 14341 | 4969 | 9372 | 10308 | 1575 | 316 | 171 | 1924 | 188090429 |
| 2004 | 14326 | 4887 | 9439 | 10185 | 1568 | 348 | 195 | 1976 | 190205384 |
| 2005 | 14519 | 4870 | 9649 | 10252 | 1622 | 327 | 181 | 2096 | 192551384 |
| 2006 | 14343 | 4920 | 9423 | 10120 | 1559 | 384 | 192 | 2050 | 195019359 |
| 2007 | 14611 | 4950 | 9661 | 10319 | 1575 | 339 | 165 | 2181 | 197403777 |
| 2008 | 15348 | 5052 | 10296 | 10800 | 1596 | 380 | 215 | 2325 | 199795090 |
| 2009 | 15632 | 5242 | 10390 | 10807 | 1722 | 380 | 249 | 2423 | 202107016 |
| 2010 | 16339 | 5481 | 10858 | 11372 | 1735 | 403 | 266 | 2502 | 203891983 |
| 2011 | 17334 | 5861 | 11473 | 11938 | 1874 | 399 | 279 | 2785 | 206592936 |
| 2012 | 18465 | 6115 | 12350 | 12742 | 1910 | 466 | 287 | 2968 | 208826037 |
| 2013 | 19806 | 6687 | 13119 | 13659 | 2082 | 465 | 311 | 3204 | 211085314 |
| 2014 | 20641 | 6862 | 13779 | 14032 | 2168 | 499 | 350 | 3481 | 213809280 |
| 2015 | 21881 | 7474 | 14407 | 14956 | 2303 | 500 | 371 | 3642 | 216553817 |
| 2016 | 23367 | 7925 | 15442 | 15994 | 2317 | 569 | 423 | 3927 | 218641417 |
| 2017 | 24855 | 8599 | 16256 | 16951 | 2456 | 640 | 467 | 4216 | 221447331 |
| 2018 | 26257 | 9043 | 17214 | 18110 | 2502 | 622 | 510 | 4399 | 223311190 |
| 2019 | 27890 | 9572 | 18318 | 19385 | 2616 | 697 | 547 | 4532 | 224981167 |
| **Total** | 374090 | 127428 | 246662 | 261210 | 39220 | 8863 | 5764 | 57485 | 4247219476 |

Supplementary Table 1: Liver Cirrhosis and Cardiovascular Disease related Deaths, Stratified by Sex and Race, in Adults in the United States, 1999 to 2019

| **Deaths** | | | | | | |
| --- | --- | --- | --- | --- | --- | --- |
| **Year** | **Medical Facility** | **Nursing Home** | **Hospices** | **Home** | **Other** | **Unknown** |
|  |  |  |  |  |  |  |
| 1999 | 8906 | 1462 | - | 2569 | 403 | - |
| 2000 | 8782 | 1489 | - | 2736 | 450 | - |
| 2001 | 8693 | 1512 | - | 2796 | 466 | - |
| 2002 | 8869 | 1522 | - | 2928 | 542 | - |
| 2003 | 8800 | 1655 | 32 | 3202 | 609 | 43 |
| 2004 | 8830 | 1624 | 57 | 3202 | 570 | 43 |
| 2005 | 8692 | 1673 | 167 | 3328 | 616 | 43 |
| 2006 | 8316 | 1713 | 217 | 3484 | 580 | 33 |
| 2007 | 8537 | 1569 | 381 | 3505 | 598 | 21 |
| 2008 | 8872 | 1609 | 409 | 3625 | 580 | 253 |
| 2009 | 8674 | 1669 | 498 | 3821 | 611 | 359 |
| 2010 | 9023 | 1737 | 611 | 4264 | 699 | - |
| 2011 | 9517 | 1835 | 727 | 4513 | 733 | - |
| 2012 | 9878 | 1972 | 917 | 4907 | 780 | 11 |
| 2013 | 10174 | 2135 | 949 | 5607 | 927 | 14 |
| 2014 | 10742 | 2158 | 1094 | 5822 | 809 | 16 |
| 2015 | 11249 | 2451 | 1350 | 6034 | 787 | 10 |
| 2016 | 11902 | 2503 | 1466 | 6659 | 833 | - |
| 2017 | 12499 | 2588 | 1725 | 7150 | 890 | - |
| 2018 | 13131 | 2693 | 1822 | 7629 | 975 | - |
| 2019 | 13672 | 2827 | 2112 | 8236 | 1036 | - |
| **Total** | 207758 | 40396 | 14534 | 96017 | 14494 | 846 |

Supplemental Table 2: Liver Cirrhosis and Cardiovascular Disease related Mortality, Stratified by Place of Death in Adults in the United States, 1999 to 2019

Supplemental Table 3 Annual percent change (APC) of Liver Cirrhosis and Cardiovascular Disease -related Age-Adjusted Mortality Rates per 100,000 in Adults in the United States, 1999 to 2019

| **Year Interval** | **APC (95% CI)** |
| --- | --- |
| **Overall** | |
| 1999-2009 | -0.5572*(-0.8086 to -0.3057) |
| 2009-2019 | 3.9588*(3.7544 to 4.1912) |
| **Male** | |
| 1999-2009 | -0.6640*(-1.003 to -0.369) |
| 2009-2019 | 3.7361*(3.4958 to 4.0233) |
| **Female** | |
| 1999-2009 | -0.6879*(-1.1747 to -0.309) |
| 2009-2019 | 4.4193*(4.1226 to 4.8392) |
| **NH White** | |
| 1999-2009 | -0.4911*(-0.9488 to -0.1181) |
| 2009-2019 | 4.5018*(4.201 to 4.9421) |
| **NH Black or African American** | |
| 1999-2008 | -2.1419*(-2.8996 to -1.5505) |
| 2008-2019 | 1.9416*(1.5672 to 2.4403) |
| **NH American Indian or Alaska Native** | |
| 1999-2007 | 0.4342(-7.8245 to 3.3753) |
| 2007-2019 | 6.3012*(5.2815 to 9.2296) |
| **NH Asian or Pacific Islander** | |
| 1999-2015 | -1.3141*(-4.3178 to -0.5439) |
| 2015-2019 | 3.4768(-0.3753 to 10.4477) |
| **Hispanic or Latino** | |
| 1999-2008 | -1.4435*(-2.0193 to -0.8644) |
| 2008-2019 | 2.0724*(1.7462 to 2.3997) |
| **Large Metropolitan areas** | |
| 1999-2009 | -1.1167*(-1.4684 to -0.8177) |
| 2009-2019 | 2.8491*(2.5981 to 3.1686) |
| **Medium- Small Metropolitan area** | |
| 1999-2009 | -0.1577(-0.7349 to 0.3284) |
| 2009-2019 | 4.8672*(4.5003 to 5.3348) |
| **Non-metropolitan area** | |
| 1999-2010 | 0.8932*(0.2718 to 1.5184) |
| 2010-2019 | 6.2255*(5.5129 to 6.9428) |
| **Northeast region** | |
| 1999-2011 | -1.3904*(-1.8525 to -1.0252) |
| 2011-2019 | 1.8922*(1.2729 to 2.8319) |
| **Midwest region** | |
| 1999-2010 | -0.5995*(-1.1701 to -0.1161) |
| 2010-2019 | 5.1173*(4.5964 to 5.823) |
| **South region** | |
| 1999-2009 | -0.5582*(-0.8998 to -0.2279) |
| 2009-2019 | 4.9266*(4.6602 to 5.2298) |
| **West region** | |
| 1999-2008 | 0.1219(-0.4483 to 0.6953) |
| 2008-2019 | 3.3094*(2.9619 to 3.658) |

APC = annual percent change; NH = non-Hispanic; * Indicates that the annual percentage change (APC) is significantly different from zero at α = 0.05. AAMR = age-adjusted mortality rate.

| **Age-Adjusted Rate (95% CI)** | | | |
| --- | --- | --- | --- |
| **Year** | **Male** | **Female** | **Overall** |
| 1999 | 10.94 (10.71 to 11.17) | 4.67 (4.54 to 4.81) | 7.54 (7.41 to 7.67) |
| 2000 | 10.81 (10.59 to 11.04) | 4.69 (4.56 to 4.83) | 7.47 (7.34 to 7.59) |
| 2001 | 10.59 (10.37 to 10.82) | 4.59 (4.45 to 4.72) | 7.32 (7.2 to 7.44) |
| 2002 | 10.63 (10.41 to 10.85) | 4.69 (4.56 to 4.83) | 7.42 (7.3 to 7.55) |
| 2003 | 10.83 (10.61 to 11.05) | 4.72 (4.59 to 4.86) | 7.53 (7.4 to 7.65) |
| 2004 | 10.63 (10.41 to 10.84) | 4.59 (4.46 to 4.72) | 7.37 (7.25 to 7.49) |
| 2005 | 10.54 (10.33 to 10.76) | 4.51 (4.39 to 4.64) | 7.32 (7.2 to 7.44) |
| 2006 | 10.07 (9.86 to 10.27) | 4.46 (4.34 to 4.59) | 7.05 (6.94 to 7.17) |
| 2007 | 10.09 (9.88 to 10.29) | 4.39 (4.27 to 4.52) | 7.05 (6.93 to 7.16) |
| 2008 | 10.47 (10.27 to 10.68) | 4.42 (4.29 to 4.54) | 7.26 (7.15 to 7.38) |
| 2009 | 10.3 (10.1 to 10.5) | 4.48 (4.36 to 4.6) | 7.21 (7.1 to 7.33) |
| 2010 | 10.59 (10.38 to 10.79) | 4.62 (4.5 to 4.75) | 7.39 (7.27 to 7.5) |
| 2011 | 10.83 (10.63 to 11.03) | 4.85 (4.72 to 4.97) | 7.65 (7.53 to 7.76) |
| 2012 | 11.43 (11.23 to 11.64) | 4.94 (4.81 to 5.07) | 8.02 (7.9 to 8.13) |
| 2013 | 11.91 (11.7 to 12.12) | 5.29 (5.17 to 5.42) | 8.39 (8.27 to 8.51) |
| 2014 | 12.18 (11.97 to 12.39) | 5.33 (5.21 to 5.46) | 8.57 (8.46 to 8.69) |
| 2015 | 12.52 (12.31 to 12.73) | 5.7 (5.57 to 5.83) | 8.88 (8.76 to 9) |
| 2016 | 13.14 (12.93 to 13.35) | 5.94 (5.8 to 6.07) | 9.32 (9.2 to 9.45) |
| 2017 | 13.61 (13.4 to 13.82) | 6.28 (6.15 to 6.42) | 9.73 (9.6 to 9.85) |
| 2018 | 14.2 (13.98 to 14.42) | 6.53 (6.39 to 6.67) | 10.08 (9.95 to 10.2) |
| 2019 | 14.8 (14.58 to 15.02) | 6.79 (6.65 to 6.93) | 10.55 (10.43 to 10.68) |
| **Total** | 11.72 (11.67 to 11.76) | 5.14 (5.11 to 5.17) | 8.2 (8.17 to 8.22) |

Supplemental Table 4 Overall and Sex‐Stratified Liver Cirrhosis and Cardiovascular Disease-related Age-Adjusted Mortality Rates per 100,000 in Adults in the United States, 1999 to 2019

| **Age-Adjusted Rate (95% CI)** | | | | | |
| --- | --- | --- | --- | --- | --- |
| **Year** | **NH White** | **NH Black or African American** | **NH American Indian or Alaska Native** | **Hispanic or Latino** | **NH Asian or Pacific Islander** |
| 1999 | 6.8 (6.67 to 6.94) | 8.8 (8.35 to 9.25) | 13.57 (11.12 to 16.03) | 14.57 (13.81 to 15.32) | 6 (5.27 to 6.73) |
| 2000 | 6.76 (6.63 to 6.9) | 8.58 (8.14 to 9.02) | 15.11 (12.6 to 17.62) | 14.71 (13.97 to 15.45) | 4.89 (4.25 to 5.54) |
| 2001 | 6.65 (6.51 to 6.78) | 8.23 (7.8 to 8.65) | 13.16 (10.92 to 15.41) | 13.81 (13.13 to 14.5) | 5.45 (4.8 to 6.1) |
| 2002 | 6.74 (6.61 to 6.88) | 8.19 (7.78 to 8.61) | 12.56 (10.4 to 14.73) | 14.01 (13.34 to 14.69) | 5.33 (4.7 to 5.96) |
| 2003 | 6.87 (6.74 to 7.01) | 8.21 (7.8 to 8.63) | 14.04 (11.8 to 16.28) | 13.99 (13.33 to 14.65) | 5.27 (4.67 to 5.88) |
| 2004 | 6.7 (6.57 to 6.84) | 7.88 (7.48 to 8.28) | 15.78 (13.42 to 18.14) | 13.58 (12.95 to 14.21) | 5.65 (5.04 to 6.27) |
| 2005 | 6.62 (6.49 to 6.75) | 7.97 (7.57 to 8.36) | 13.89 (11.78 to 16.01) | 13.7 (13.08 to 14.32) | 4.94 (4.38 to 5.5) |
| 2006 | 6.46 (6.33 to 6.58) | 7.44 (7.06 to 7.82) | 14.92 (12.68 to 17.15) | 12.87 (12.28 to 13.46) | 5.37 (4.81 to 5.93) |
| 2007 | 6.47 (6.34 to 6.59) | 7.29 (6.92 to 7.66) | 12.43 (10.41 to 14.45) | 12.94 (12.36 to 13.51) | 4.62 (4.11 to 5.13) |
| 2008 | 6.65 (6.52 to 6.77) | 7.13 (6.77 to 7.49) | 15.5 (13.32 to 17.68) | 13.05 (12.49 to 13.61) | 4.99 (4.47 to 5.51) |
| 2009 | 6.55 (6.43 to 6.68) | 7.43 (7.07 to 7.79) | 17.22 (14.97 to 19.47) | 13.12 (12.57 to 13.67) | 4.66 (4.18 to 5.15) |
| 2010 | 6.8 (6.68 to 6.93) | 7.42 (7.06 to 7.78) | 18.29 (15.99 to 20.59) | 13.02 (12.48 to 13.56) | 4.74 (4.26 to 5.22) |
| 2011 | 7.02 (6.89 to 7.15) | 7.65 (7.29 to 8) | 19.02 (16.67 to 21.37) | 13.48 (12.96 to 14.01) | 4.35 (3.9 to 4.79) |
| 2012 | 7.42 (7.28 to 7.55) | 7.61 (7.26 to 7.96) | 19 (16.7 to 21.3) | 13.94 (13.41 to 14.47) | 4.84 (4.39 to 5.3) |
| 2013 | 7.83 (7.7 to 7.97) | 8.12 (7.76 to 8.48) | 19.53 (17.29 to 21.77) | 14.37 (13.85 to 14.89) | 4.63 (4.2 to 5.06) |
| 2014 | 7.93 (7.79 to 8.06) | 8.19 (7.84 to 8.55) | 21.6 (19.25 to 23.95) | 14.59 (14.09 to 15.1) | 4.67 (4.25 to 5.09) |
| 2015 | 8.31 (8.17 to 8.45) | 8.51 (8.16 to 8.87) | 21.77 (19.48 to 24.05) | 14.81 (14.31 to 15.31) | 4.34 (3.95 to 4.73) |
| 2016 | 8.75 (8.61 to 8.89) | 8.29 (7.95 to 8.64) | 24.66 (22.23 to 27.1) | 15.43 (14.92 to 15.93) | 4.78 (4.38 to 5.18) |
| 2017 | 9.2 (9.05 to 9.34) | 8.69 (8.34 to 9.05) | 26.66 (24.16 to 29.16) | 15.67 (15.17 to 16.16) | 5.18 (4.77 to 5.59) |
| 2018 | 9.69 (9.54 to 9.83) | 8.68 (8.33 to 9.02) | 28.84 (26.26 to 31.42) | 15.81 (15.32 to 16.29) | 4.75 (4.37 to 5.13) |
| 2019 | 10.24 (10.09 to 10.39) | 8.82 (8.47 to 9.17) | 30.26 (27.64 to 32.87) | 15.65 (15.18 to 16.12) | 5.12 (4.73 to 5.51) |
| **Total** | 7.57 (7.54 to 7.6) | 8.18 (8.1 to 8.27) | 19.2 (18.69 to 19.72) | 14.36 (14.24 to 14.49) | 4.94 (4.83 to 5.04) |

Supplemental Table 5: Race‐Stratified Liver Cirrhosis and Cardiovascular Disease -related Age-Adjusted Mortality Rates per 100,000 in Adults in the United States, 1999 to 2020

NH: Non-Hispanic

Supplemental Table 6: Liver Cirrhosis and Cardiovascular Disease Disease-Related Age-Adjusted Mortality Rates per 100,000, Stratified by States in Adults in the United States, 1999 to 2019

| **State** | **Age-Adjusted Rate (95% CI)** |
| --- | --- |
| Alabama | 7.54 (7.34 to 7.74) |
| Alaska | 6.18 (5.62 to 6.75) |
| Arizona | 7.78 (7.59 to 7.96) |
| Arkansas | 7.1 (6.85 to 7.35) |
| California | 13.42 (13.32 to 13.52) |
| Colorado | 7.19 (6.99 to 7.4) |
| Connecticut | 7.49 (7.26 to 7.72) |
| Delaware | 6.55 (6.12 to 6.97) |
| District of Columbia | 8.26 (7.64 to 8.88) |
| Florida | 7.43 (7.34 to 7.53) |
| Georgia | 6.87 (6.73 to 7.02) |
| Hawaii | 7.92 (7.54 to 8.3) |
| Idaho | 5.76 (5.43 to 6.08) |
| Illinois | 5.52 (5.41 to 5.62) |
| Indiana | 7.12 (6.95 to 7.29) |
| Iowa | 5.29 (5.08 to 5.49) |
| Kansas | 5.57 (5.34 to 5.79) |
| Kentucky | 8.35 (8.13 to 8.57) |
| Louisiana | 6.61 (6.42 to 6.81) |
| Maine | 5.61 (5.3 to 5.92) |
| Maryland | 6.3 (6.13 to 6.47) |
| Massachusetts | 6.54 (6.38 to 6.7) |
| Michigan | 6.59 (6.46 to 6.72) |
| Minnesota | 6.44 (6.26 to 6.62) |
| Mississippi | 9.25 (8.96 to 9.54) |
| Missouri | 5.22 (5.07 to 5.37) |
| Montana | 5.79 (5.41 to 6.17) |
| Nebraska | 6.2 (5.9 to 6.5) |
| Nevada | 8.61 (8.31 to 8.91) |
| New Hampshire | 6.66 (6.3 to 7.01) |
| New Jersey | 6.27 (6.14 to 6.41) |
| New Mexico | 10.24 (9.87 to 10.61) |
| New York | 8.66 (8.55 to 8.77) |
| North Carolina | 7.25 (7.11 to 7.39) |
| North Dakota | 6.51 (6.01 to 7.01) |
| Ohio | 8.27 (8.13 to 8.4) |
| Oklahoma | 10.8 (10.53 to 11.08) |
| Oregon | 7.24 (7.02 to 7.46) |
| Pennsylvania | 6.67 (6.56 to 6.78) |
| Rhode Island | 11.31 (10.8 to 11.83) |
| South Carolina | 8.91 (8.69 to 9.13) |
| South Dakota | 6.32 (5.86 to 6.77) |
| Tennessee | 9.33 (9.13 to 9.52) |
| Texas | 11.63 (11.51 to 11.75) |
| Utah | 4.27 (4.03 to 4.5) |
| Vermont | 8.04 (7.49 to 8.6) |
| Virginia | 5.46 (5.32 to 5.59) |
| Washington | 8.07 (7.9 to 8.25) |
| West Virginia | 10.19 (9.83 to 10.54) |
| Wisconsin | 5.59 (5.43 to 5.75) |
| Wyoming | 6.56 (5.99 to 7.13) |

Supplemental Table 7: Liver Cirrhosis and Cardiovascular Disease –related Age-Adjusted Mortality Rates per 100,000, Stratified by Census Region in Adults in the United States, 1999 to 2019

| **Census Region** | **Year** | **Age-Adjusted Rate (95% CI)** |
| --- | --- | --- |
| Northeast | 1999 | 8.03 (7.74 to 8.32) |
| Northeast | 2000 | 8 (7.71 to 8.29) |
| Northeast | 2001 | 7.76 (7.48 to 8.05) |
| Northeast | 2002 | 7.7 (7.42 to 7.98) |
| Northeast | 2003 | 7.66 (7.38 to 7.94) |
| Northeast | 2004 | 7.57 (7.29 to 7.84) |
| Northeast | 2005 | 7.27 (7 to 7.54) |
| Northeast | 2006 | 6.98 (6.72 to 7.24) |
| Northeast | 2007 | 7.05 (6.79 to 7.31) |
| Northeast | 2008 | 7.12 (6.86 to 7.39) |
| Northeast | 2009 | 6.89 (6.63 to 7.14) |
| Northeast | 2010 | 6.98 (6.73 to 7.24) |
| Northeast | 2011 | 6.68 (6.43 to 6.93) |
| Northeast | 2012 | 7.12 (6.86 to 7.37) |
| Northeast | 2013 | 7.16 (6.9 to 7.41) |
| Northeast | 2014 | 7.06 (6.81 to 7.31) |
| Northeast | 2015 | 7.15 (6.9 to 7.41) |
| Northeast | 2016 | 7.6 (7.34 to 7.85) |
| Northeast | 2017 | 7.56 (7.31 to 7.82) |
| Northeast | 2018 | 7.74 (7.49 to 8) |
| Northeast | 2019 | 7.82 (7.57 to 8.08) |
| Northeast | **Total** | 7.41 (7.35 to 7.47) |
| Midwest | 1999 | 5.98 (5.74 to 6.22) |
| Midwest | 2000 | 5.68 (5.45 to 5.91) |
| Midwest | 2001 | 5.7 (5.48 to 5.93) |
| Midwest | 2002 | 5.99 (5.76 to 6.22) |
| Midwest | 2003 | 5.94 (5.71 to 6.17) |
| Midwest | 2004 | 5.76 (5.53 to 5.98) |
| Midwest | 2005 | 5.69 (5.47 to 5.91) |
| Midwest | 2006 | 5.44 (5.22 to 5.65) |
| Midwest | 2007 | 5.27 (5.06 to 5.48) |
| Midwest | 2008 | 5.64 (5.43 to 5.86) |
| Midwest | 2009 | 5.59 (5.37 to 5.8) |
| Midwest | 2010 | 5.54 (5.33 to 5.75) |
| Midwest | 2011 | 5.85 (5.63 to 6.06) |
| Midwest | 2012 | 6.21 (5.99 to 6.43) |
| Midwest | 2013 | 6.5 (6.27 to 6.72) |
| Midwest | 2014 | 6.69 (6.46 to 6.92) |
| Midwest | 2015 | 6.97 (6.74 to 7.2) |
| Midwest | 2016 | 7.37 (7.14 to 7.61) |
| Midwest | 2017 | 7.81 (7.57 to 8.05) |
| Midwest | 2018 | 8.18 (7.93 to 8.42) |
| Midwest | 2019 | 8.73 (8.48 to 8.98) |
| Midwest | **Total** | 6.43 (6.38 to 6.48) |
| South | 1999 | 7.25 (7.04 to 7.47) |
| South | 2000 | 7.37 (7.16 to 7.58) |
| South | 2001 | 7.21 (7 to 7.41) |
| South | 2002 | 7.27 (7.06 to 7.47) |
| South | 2003 | 7.41 (7.21 to 7.62) |
| South | 2004 | 7.15 (6.95 to 7.35) |
| South | 2005 | 7.23 (7.03 to 7.43) |
| South | 2006 | 6.86 (6.67 to 7.05) |
| South | 2007 | 7 (6.81 to 7.19) |
| South | 2008 | 6.96 (6.77 to 7.14) |
| South | 2009 | 7.17 (6.98 to 7.35) |
| South | 2010 | 7.31 (7.13 to 7.5) |
| South | 2011 | 7.69 (7.5 to 7.88) |
| South | 2012 | 8.01 (7.82 to 8.2) |
| South | 2013 | 8.41 (8.22 to 8.61) |
| South | 2014 | 8.77 (8.58 to 8.97) |
| South | 2015 | 9.21 (9.01 to 9.4) |
| South | 2016 | 9.67 (9.47 to 9.87) |
| South | 2017 | 10.34 (10.13 to 10.55) |
| South | 2018 | 10.8 (10.59 to 11.01) |
| South | 2019 | 11.3 (11.09 to 11.51) |
| South | **Total** | 8.3 (8.26 to 8.35) |
| West | 1999 | 9.17 (8.86 to 9.48) |
| West | 2000 | 9.2 (8.9 to 9.51) |
| West | 2001 | 8.98 (8.68 to 9.28) |
| West | 2002 | 8.95 (8.65 to 9.24) |
| West | 2003 | 9.3 (9 to 9.6) |
| West | 2004 | 9.3 (9.01 to 9.6) |
| West | 2005 | 9.21 (8.92 to 9.5) |
| West | 2006 | 9.18 (8.9 to 9.47) |
| West | 2007 | 9.01 (8.73 to 9.29) |
| West | 2008 | 9.42 (9.13 to 9.7) |
| West | 2009 | 9.15 (8.88 to 9.43) |
| West | 2010 | 9.69 (9.41 to 9.97) |
| West | 2011 | 10.23 (9.94 to 10.51) |
| West | 2012 | 10.46 (10.18 to 10.74) |
| West | 2013 | 11.31 (11.02 to 11.6) |
| West | 2014 | 11.24 (10.95 to 11.53) |
| West | 2015 | 11.62 (11.33 to 11.91) |
| West | 2016 | 11.96 (11.67 to 12.25) |
| West | 2017 | 12.32 (12.03 to 12.61) |
| West | 2018 | 12.63 (12.34 to 12.92) |
| West | 2019 | 13.11 (12.82 to 13.41) |
| West | **Total** | 10.47 (10.41 to 10.54) |

Supplemental Table 8: Liver Cirrhosis and Cardiovascular Disease – related Age-Adjusted Mortality Rates per 100,000, Stratified by Urbanization in Adults in the United States, 1999 to 2019

|  | **Age-Adjusted Rate (95% CI)** | | |
| --- | --- | --- | --- |
| **Year** | Large Metropolitan | Medium-Small metropolitan | Non-metropolitan |
| 1999 | 8.11 (7.93 to 8.29) | 7.17 (6.95 to 7.4) | 6.42 (6.14 to 6.7) |
| 2000 | 8.01 (7.83 to 8.19) | 7.26 (7.03 to 7.48) | 6.35 (6.07 to 6.62) |
| 2001 | 7.62 (7.44 to 7.79) | 7.29 (7.07 to 7.52) | 6.68 (6.4 to 6.96) |
| 2002 | 7.8 (7.62 to 7.97) | 7.23 (7.01 to 7.45) | 6.61 (6.33 to 6.89) |
| 2003 | 7.82 (7.65 to 7.99) | 7.46 (7.23 to 7.68) | 6.84 (6.55 to 7.12) |
| 2004 | 7.75 (7.58 to 7.93) | 7.1 (6.88 to 7.31) | 6.75 (6.47 to 7.03) |
| 2005 | 7.54 (7.37 to 7.71) | 7.24 (7.02 to 7.45) | 6.83 (6.55 to 7.11) |
| 2006 | 7.28 (7.12 to 7.45) | 7 (6.79 to 7.21) | 6.6 (6.33 to 6.87) |
| 2007 | 7.18 (7.02 to 7.34) | 7.1 (6.89 to 7.31) | 6.52 (6.25 to 6.79) |
| 2008 | 7.31 (7.15 to 7.47) | 7.31 (7.1 to 7.52) | 7 (6.72 to 7.27) |
| 2009 | 7.19 (7.03 to 7.35) | 7.26 (7.05 to 7.47) | 7.18 (6.9 to 7.45) |
| 2010 | 7.43 (7.27 to 7.59) | 7.4 (7.19 to 7.61) | 7.36 (7.07 to 7.64) |
| 2011 | 7.54 (7.38 to 7.7) | 7.83 (7.62 to 8.04) | 7.8 (7.51 to 8.08) |
| 2012 | 7.84 (7.68 to 8) | 8.22 (8 to 8.43) | 8.1 (7.81 to 8.39) |
| 2013 | 8.21 (8.05 to 8.37) | 8.9 (8.68 to 9.12) | 8.25 (7.96 to 8.55) |
| 2014 | 8.36 (8.2 to 8.52) | 8.82 (8.6 to 9.04) | 8.76 (8.46 to 9.06) |
| 2015 | 8.49 (8.33 to 8.65) | 9.46 (9.23 to 9.68) | 9.29 (8.98 to 9.6) |
| 2016 | 8.81 (8.65 to 8.97) | 9.94 (9.71 to 10.17) | 9.99 (9.67 to 10.32) |
| 2017 | 8.94 (8.78 to 9.1) | 10.71 (10.48 to 10.95) | 10.66 (10.32 to 10.99) |
| 2018 | 9.24 (9.08 to 9.41) | 10.88 (10.65 to 11.12) | 11.82 (11.47 to 12.17) |
| 2019 | 9.48 (9.31 to 9.64) | 11.51 (11.27 to 11.75) | 12.46 (12.1 to 12.82) |
| Total | 8.09 (8.06 to 8.13) | 8.42 (8.37 to 8.47) | 8.14 (8.07 to 8.2) |
